# Intense inflammation in bladder carcinoma is associated with angiogenesis and indicates good prognosis

**DOI:** 10.1038/sj.bjc.6600615

**Published:** 2002-12-02

**Authors:** B V Offersen, M M Knap, N Marcussen, M R Horsman, S Hamilton-Dutoit, J Overgaard

**Affiliations:** Danish Cancer Society, Department of Experimental Clinical Oncology, Aarhus University Hospital, Noerrebrogade 44, bldg 5, DK-8000 Aarhus C, Denmark; Institute of Pathology, Aarhus University Hospital, Noerrebrogade 44, bldg 5, DK-8000 Aarhus C, Denmark

**Keywords:** bladder carcinoma, angiogenesis, inflammation, prognosis

## Abstract

The aim of this study was to investigate the prognostic influence of microvessel density using the hot spot method in 107 patients diagnosed with transitional cell carcinoma of the bladder. In each case, inflammation was found in the invasive carcinoma, therefore we classified the degree of inflammation as minimal, moderate or intense. Microvessel density was then reevaluated in each tumour in areas corresponding to these three categories. Median microvessel density irrespective of degree of inflammation was 71. Areas of minimal, moderate and intense inflammation were found in 48, 92 and 32 tumours. Microvessel density increased significantly with increasing degree of inflammation. Disease-specific survival was improved if areas of intense inflammation were present in the carcinoma (*P*=0.004). High microvessel density, irrespective of the degree of inflammation, was associated with a significantly better disease-specific survival (*P*=0.01). Multivariate analysis using death of disease as endpoint demonstrated an independent prognostic value of N-classification (N0, hazard ratio (HR)=1 *vs* N1, HR=2.89 (range, 1.52–5.52) *vs* N2, HR=3.61 (range, 1.84–7.08)), and intense inflammation, HR=0.48 (range, 0.24–0.96). Malignancy grade, T classification and microvessel density were not independent significant markers of poor outcome. In conclusion, inflammation was significantly correlated to microvessel density, and areas of intense inflammation were an independent marker of good prognosis.

*British Journal of Cancer* (2002) **87**, 1422–1430. doi:10.1038/sj.bjc.6600615 www.bjcancer.com

© 2002 Cancer Research UK

## 

Angiogenesis, the development of new vessels from pre-existing vessels, is involved in the growth, maintenance and metastasis of most solid tumours (Zetter, 1988). Several groups have investigated the prognostic significance of estimates of tumour angiogenesis in bladder carcinoma. Although these studies (Dickinson *et al*, 1994; Bochner *et al*, 1995; Jaeger *et al*, 1995; Philp *et al*, 1996; Chaudhary *et al*, 1999; Inoue *et al*, 2000) used slightly different methods, they support a theory that a highly vascular bladder carcinoma behaves more aggressively than a carcinoma with a low vascular density. The development of new vessels is influenced by pro-angiogenic factors, vascular endothelial growth factor (VEGF) being one of the most potent stimulators of angiogenesis (Ferrara, 1999). Carcinoma cells, plasma cells, and T lymphocytes have been shown to synthesise VEGF (Freeman *et al*, 1995; Ito *et al*, 1995), implying that inflammation can influence angiogenesis, but no agreed guidance exists on how to deal with inflammation when estimating angiogenesis. To our knowledge no studies on estimates of angiogenesis in relation to inflammation have been published.

Inflammation is a frequent finding in bladder carcinoma and may be of prognostic significance. In a study on 428 cases of primary superficial bladder carcinoma, patients with an inflammatory reaction in their tumour experienced significantly fewer recurrences and cancer-related deaths compared to those with tumours without inflammation (Flamm, 1992). Another study on 56 patients with invasive bladder carcinoma reported a significantly better 1-year survival when lymphocytes, plasma cells and/or lymph follicles were present in the tumours (Mihatsch *et al*, 1979). These findings suggest a protective role of inflammation. The possible positive influence of toxins on cancer has been known for many years. Indeed, Coley's observations in 1893 initiated studies on the possible anti-cancer effects of Coley's toxin (Coley, 1893). The cure of a patient with recurrent lymphosarcoma after two erysipelas infections prompted Coley to use filtered bacterial extracts (Coley's toxin) for adjuvant carcinoma therapy. More recently, use has been made of the anti-tumour effects of inflammation by inducing a local immune response to BCG (bacillus of Calmette and Guerin) instillations into the bladder in patients with urothelial tumours. The precise mechanisms involved in this process are still to be clarified (Alexandroff *et al*, 1999; Bohle, 2000). In 1986, Jass classified inflammation in the invasive part of adenocarcinomas of the rectum as pronounced, moderate, and little or none (Jass, 1986). He found that high degree of inflammation in close association with the invasive carcinoma was an independent parameter of good prognosis.

In the present study, we investigated the prognostic impact of estimates of angiogenesis in invasive bladder carcinoma. Our initial studies showed an unexpectedly high frequency of inflammation associated with the invasive tumour component. This prompted us to analyse angiogenesis in relation to the degree of inflammation, and to correlate these estimates with other known prognostic factors, disease-specific and overall survival.

## MATERIALS AND METHODS

### Patients

Based on cystoscopy and biopsy 107 consecutive patients diagnosed with transitional cell carcinoma of the bladder were considered candidates for radical cystectomy at the University Hospital of Aarhus, Denmark, between January 1, 1992 and December 31, 1998. The study was carried out with ethical committee approval. Tumour, Nodal, Metastasis (TNM) classifications were based on the guidelines of the International Union against Cancer (UICC), 1992, and morphological classification and grading of the tumours were performed according to Bergkvist *et al* (1965) by one uropathologist. Formalin-fixed and paraffin-embedded surgical specimens of invasive tumour tissue coming from transurethral resection of the bladder (TUR-B) were used in the present study. The characteristics of the patients are listed in Table 1

**Table 1:**
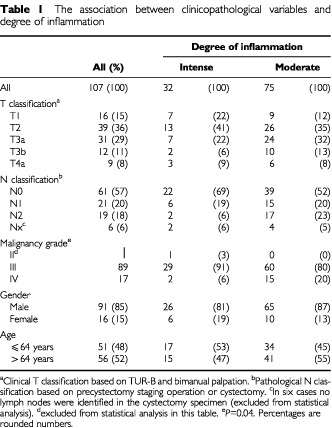
The association between clinicopathological variables and degree of inflammation

with regard to sex, age, clinical T classification (based on TUR-B and bimanual palpation), pathological N classification (based on findings during precystectomy staging operation or cystectomy), and histopathological grade. In six cases no lymph nodes were identified in the cystectomy specimen. Following evaluation by chest X-ray, excretory urogram, bone scintigraphy and either ultrasound, CT, or MR, all tumours were classified as M0. Eight cases (50%) with T1 had CIS in the TUR-B specimen.

Eight patients (7%) had previous BCG instillations into the bladder. Eighty-seven patients (81%), mainly with muscle-invasive tumours, underwent a pre-cystectomy staging operation with uni- or bilateral pelvic lymphadenectomy. Based on this, 40 patients (37%) (three N_0_, 19 N_1_, 18 N_2_) were treated with radiotherapy (60 Gy) and/or chemotherapy, and 67 patients (63%) underwent radical cystectomy. All time estimates were made using the date of TUR-B just before staging and/or cystectomy as the initial value, and the patients were followed until death or August 1, 2001. At analysis, 60 patients (56%) had died from bladder carcinoma, and eight patients (7%) had died with no evidence of disease. Median follow-up was 33 months (range, 4–85 months). Median time to death for the 60 patients who died from bladder carcinoma was 18 months (range, 4–67 months). No patients were lost to follow-up or omitted from the analyses for other reasons.

### Microvessel staining

In 81 cases (76%) one paraffin block from the TUR-B contained tumour, whereas two, three, and four tumour containing paraffin blocks were available in 18 (17%), six (5%), and two cases (2%), respectively. When more than one block was available we used the same paraffin block as had been used previously by the pathologist for histopathological diagnosis. Each case was represented by a single section. Sections were mounted on silanised glass slides and stored in the dark at 5°C before staining within 1 week. After removal of paraffin and rehydration, tissue sections underwent antigen retrieval by microwave heating in 10 mM Tris and 0.5 mM EGTA buffer (Titriplex® VI, Merck Eurolab, Copenhagen, Denmark) (pH 9.0) twice for 5 min. Endogeneous peroxidase was blocked by incubation in 5% H_2_O_2_ in distilled water for 20 min at room temperature, before overnight incubation at 4°C with an anti-CD34 monoclonal antibody (clone QB-END 10, Immunotech, Marseille, France) diluted 1 : 200 in Antibody Diluent (S809, Dako, Glostrup, Denmark) to label endothelial cells. Bound primary antibody was detected by the application of Envision+™ (Dako Envision+™, Peroxidase, Mouse, K4001). Intervening washes were performed in Tris/PBS for 5 min. The antigen-antibody complex was visualised using the chromogen 3-amino-9-ethylcarbazole (a-5754, Sigma-Aldrich, Copenhagen, Denmark), counterstained with Mayer's haematoxylin and mounted in DAKO Glycergel (code No. C 0563).

### Microvessel quantification

Tumours were scanned at ×40–100 magnification for areas in the invasive carcinomas demonstrating the highest microvascular density (MVD_max_) and estimates of angiogenesis were made using a 10×10 grid in the ocular projected onto this area using a slight modification of the method first described by Weidner *et al* (1991) and used by us previously (Offersen *et al*, 2001). The counts were done at ×200 magnification, area 0.25 mm^2^. All sections showed adequate staining. All the counts were done without knowledge of patient outcome by a single observer (BVO).

### Evaluation of inflammation

During initial measurements of MVD_max_, inflammation within the invasive component of the carcinomas was noted to be far more intense than we had encountered previously when estimating angiogenesis in carcinomas of the prostate, lung and breast. Therefore, we established a semiquantitative scale to assess the degree of inflammation, which to our knowledge has not been described in bladder carcinoma. Inflammation was scored as: minimal inflammation (less than five lymphocytes within the 10×10 grid at ×200, area 0.25 mm^2^); moderate inflammation (mononuclear inflammatory cells scattered throughout the tissue but background stromal connective tissue still clearly visible); intense inflammation (mononuclear inflammatory cells densely infiltrating the tissues lying side-by-side). The degree of inflammation in the individual tumours was assessed throughout the invasive carcinoma component. Although lymphoid follicles with germinal centres were encountered these were not separately assessed. Also, no evaluations were done in areas of necrosis. MVD in areas of minimal, moderate or intense inflammation were designated MVD1, MVD2, and MVD3, respectively. The reproducibility of identifying the different degrees of inflammation was tested in 20 randomly chosen tumours. Estimates of MVD in areas of the three different degrees of inflammation were done on three separate occasions. Sections from 10 randomly chosen tumours were blindly recounted to test reproducibility of the MVD scores.

### VEGF immunohistochemical assay

Parallel sections to the anti-CD34 stained slides were processed as described above, except for being microwaved in buffer for 3×5 min. They were then incubated with anti-VEGF (sc152, rabbit polyclonal IgG, 200 μg ml^−1^; Santa Cruz Biotechnology, CA, USA) diluted 1 : 2000 in Antibody Diluent (Dako) overnight at 4°C. This antibody reacts with the 121, 165, and 189 amino acid splice variants of VEGF of human origin. Bound primary antibody was detected by Dako Envision+™ (peroxidase, rabbit, code no. K4003) for 30 min, followed by 7 min incubation in Vector® NovaRED™ (Substrate kit for peroxidase, SK-4800, Vector Laboratories, Burlingame, CA, USA). Slides were counterstained with Mayer's haematoxylin, and mounted in a non-aqueous media (DPX, BDH Laboratory Supplies, Poole, UK). Intervening washes were performed in Tris/PBS (pH 7.6) with added 0.25% Triton X-100. The positive control was a section of prostate carcinoma previously shown to have a high VEGF content by immunohistochemistry. VEGF staining was considered positive if appropriate red staining was seen in the cell cytoplasm.

### Statistical methods

The reproducibility of the MVD method was tested in accordance to the guidelines suggested by Bland and Altman (1986), thus scatter plots and difference plots were made to graphically show the reproducibility. MVD1 and MVD2 were compared with a Mann–Whitney *U*-test, because although both counts were normally distributed, a *F*-test showed significantly higher variances in the inflammatory group (MVD2). MVD2 and MVD3 were compared with a *t*-test. A χ^2^ test was used when looking for a correlation between the different vascular estimates dichotomised by their median values and other known clinicopathological parameters. Survival functions were performed according to the Kaplan–Meier method, differences in survival curves being calculated according to the log-rank test. A multivariate Cox proportional hazards regression analysis was used to investigate the prognostic value of the clinicopathological parameters with respect to death of disease or overall death applying a backward LR test. Univariate and Cox multivariate analyses were done using the SPSS 10.0 program package. All *P*-values were based on two-sided testing and the level of statistical significance was 5%.

## RESULTS

### MVDmax

**Figure 1:**
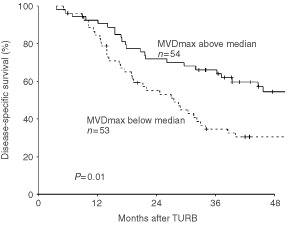
Disease-specific survival stratified by MVD_max_ evaluated irrespective of degree of inflammation dichotomised on the median value (MVD_max_ high ⩾71).

**Figure 2:**
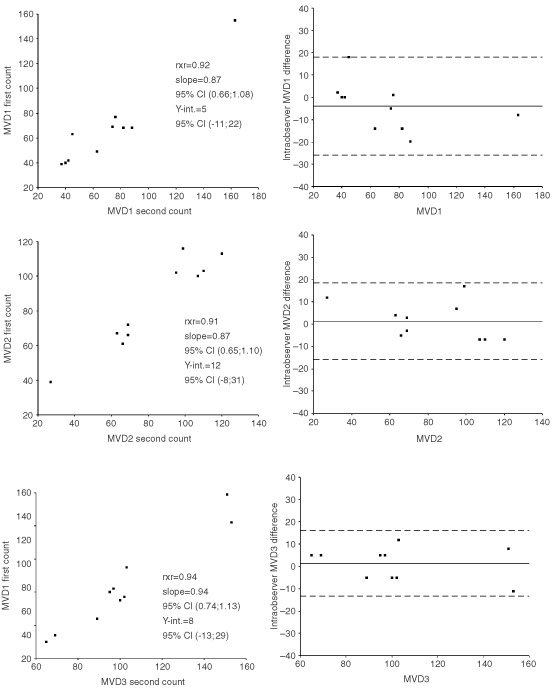
Intraobserver data for the reproducibility of the MVD counts. Left column shows the bivariate scatter plots. In each plot the correlation coefficient r^2^, slope with corresponding 95% confidence interval, and intercept with the *Y*-axis with corresponding 95% confidence interval are listed. Right column shows the difference plots; solid line : mean intraobserver difference and dotted line : 95% limits of agreement (±2 s.d.).

Median MVD_max_ was 71 (range, 21–249). MVD_max_ dichotomised by the median value was independent of the clinicopathological factors mentioned in Table 1, i.e. T and N classification, malignancy grade, gender and age (data not shown). Furthermore, MVD_max_ dichotomised by the median value is shown in Figure 1

and illustrates a significantly better disease-specific survival of the patients whose tumours had a vascular score ⩾71 (*P*=0.01). Reproducibility was acceptable, as all the recounts were highly correlated in the scatter plots and were situated between the lines of ±2 s.d. in Figure 2

.

### Inflammation

All of the 107 carcinomas had areas of inflammation in their invasive component, but the degree varied, and indeed, most of the tumours had at least two different degrees of inflammation in different areas of the invasive carcinoma. Table 2

**Table 2:**
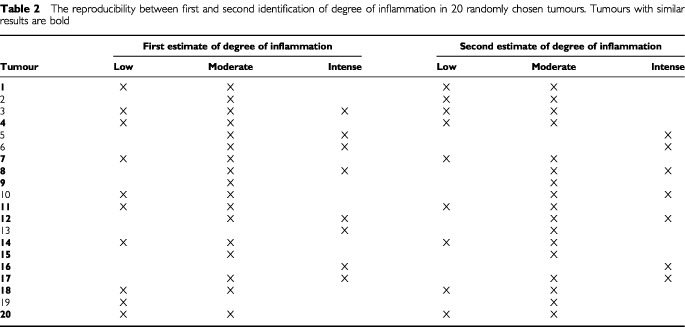
The reproducibility between first and second identification of degree of inflammation in 20 randomly chosen tumours. Tumours with similar results are bold

demonstrates the reproducibility of identifying the different degrees of inflammation in the carcinomas. In 13 (65%) tumours the inflammatory scores were identical. Nine of the tumours had areas of intense inflammation identified either in the first or the second round, and in six (2 out of 3) of these tumours the first and second scores were similar.

**Figure 3:**
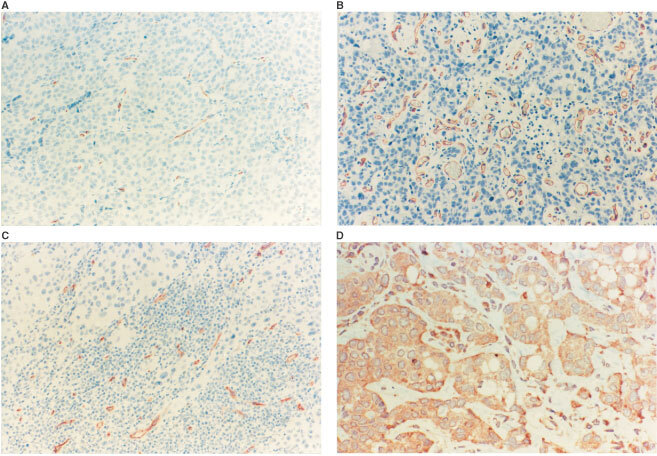
(**A**–**C**) Shows examples of minimal, moderate and intense inflammation, respectively. CD34-positive vessels are highlighted with red and counterstained with haematoxylin. Notice in **C** carcinoma cells lying in close relation to the inflammatory cells. (**D**) Shows VEGF staining of an adjacent section to **B**. Notice the intense staining of inflammatory cells in the stroma and of the carcinoma cells. Bar is 100 μm.

**Figure 4:**
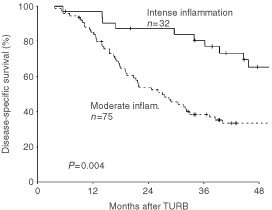
Disease-specific survival stratified by tumours with areas of intense and moderate inflammation in bladder carcinoma.

Forty-eight of the tumours (45%) had areas in the invasive carcinoma with minimal associated inflammation (Figure 3A

). However, in each of these 48 tumours, areas of moderate inflammation were also present in other parts of the carcinoma. In another 44 tumours, areas of moderate inflammation were present in the carcinoma, and these tumours had no areas of minimal inflammation. Thus, in a total of 92 tumours (86%) (Figure 3B) areas of moderate inflammation were present. Areas of intense inflammation were encountered in 32 cases (30%) (Figure 3C), 15 being intensely inflamed throughout the carcinoma, nine also showing areas with moderate inflammation, and eight showing areas with all three degrees of inflammation. Table 1 shows that the degrees of inflammation were independent of other known clinico-pathological variables, except malignancy grade. The prognostic value of inflammation was then investigated, and the tumours were separated in those with areas of intense inflammation as compared to those with areas of moderate inflammation (Figure 4

). The survival plot showed a significantly better disease-specific survival if areas of intense inflammation were present in the carcinoma (*P*=0.004), with the estimated 4 year disease-specific actuarial survival rate being 65% if intense inflammation was present in invasive bladder carcinoma compared to 34% when it was not present.

### MVD and VEGF in areas of inflammation

**Figure 5:**
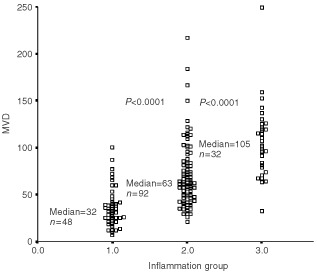
Distribution of MVD evaluated in areas of different degrees of inflammation. Inflammation group 1 was ‘minimal inflammation’, group 2 was ‘moderate inflammation’, and group 3 was ‘intense inflammation’.

The median value of MVD1 was 32 (range, 7–100), median MVD2 was 63 (range, 21–217), and median MVD3 was 105 (range, 32–249) (Figure 5

). The vascular scores increased significantly with increasing degree of inflammation (*P*<0.0001). Neither of the vascular scores could separate the clinicopathological variables shown in Table 1 indicating that the vascular scores were independent parameters. VEGF staining showed in all but a few cases that the staining intensity was very strong in both the carcinoma cells and the non-neoplastic cells, Figure 3D.

### Multivariate analysis

A Cox multivariate analysis was performed using death from bladder carcinoma as the endpoint. As N status was uncertain in six cases, we omitted these patients from the multivariate analysis, which therefore was performed in 101 patients with all variables available. Table 3

**Table 3:**
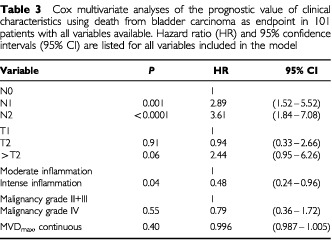
Cox multivariate analyses of the prognostic value of clinical characteristics using death from bladder carcinoma as endpoint in 101 patients with all variables available. Hazard ratio (HR) and 95% confidence intervals (95% CI) are listed for all variables included in the model

shows all the variables included in the model. Notice, that in this model, degree of inflammation was an independent variable, whereas MVD_max_ was not independent. However, if degree of inflammation was omitted from the model, MVD_max_ evaluated as a continuous parameter was almost an independent prognostic parameter (HR=0.992, 95% CI 0.984–1.001, *P*=0.08). Using overall death as endpoint in the multivariate analysis showed the close association between degree of inflammation and MVD_max_, since MVD_max_ now was an independent variable, while degree of inflammation lost importance (Table 4

**Table 4:**
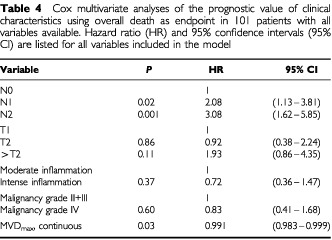
Cox multivariate analyses of the prognostic value of clinical characteristics using overall death as endpoint in 101 patients with all variables available. Hazard ratio (HR) and 95% confidence intervals (95% CI) are listed for all variables included in the model

).

## DISCUSSION

We investigated the potential prognostic value of microvascular density in invasive bladder carcinoma. During our initial attempts to count MVD_max_, inflammation was identified as a complicating factor. Therefore, we established a semiquantitative score of inflammation in relation to which vascular densities could be evaluated. Our analysis showed significantly increased vascular density with increasing degree of inflammation, and identified areas of intense inflammation in the invasive carcinoma as a new independent indicator of good prognosis. Estimates of angiogenesis were found to be associated with inflammation, since the vascular densities increased with increasing degree of inflammation. Both carcinoma and non-neoplastic cells were identified as contributors of VEGF in the tissues implying that both cell types may stimulate angiogenic processes. The predominant inflammatory cell types found in the tumours in the present study were lymphocytes and plasma cells reflecting a chronic inflammatory response to the tumour, but polymorphonuclear cells (PMN) were also encountered reflecting signs of acute inflammation. Recently it has been demonstrated that activated PMNs can release VEGF from intracellular stores and thereby stimulate angiogenesis (Mccourt *et al*, 1999).

The presence of inflammation was evaluated in one histological section from the same paraffin block which had been used for diagnostical histopathological evaluation in each patient. This was selected by the pathologist as the one containing most representative carcinoma. Others have investigated the intratumour variability of MVD estimates in breast carcinomas, and found that the largest contributor to total variation was the intertumour variation (de Jong *et al*, 1995). The variation among sections from the same tumour block was evaluated and compared to the variation among different tumour blocks from the same tumour and between tumours, and de Jong *et al* (1995) reported ‘that variation between blocks contributed most to the total variation (60%), followed by variation within blocks (34%) and sections (6%)’. Furthermore, they compared MVD evaluated by the hot spot method with MVD evaluated with a systematic random technique, and concluded: ‘When comparing the systematic counts with the hot spot counts, the relatively low variation within blocks speaks in favour of the hot spot method, because this implies that from available blocks, only one section has to be scanned to find the hot spot’. On the other hand, considerable variation in MVD estimates within the same paraffin block has recently been shown in another study (Ahlgren *et al*, 2002). In the present study we have not investigated the variability of degree of inflammation in single paraffin blocks, nor among paraffin blocks from the same tumour.

Five other studies estimating angiogenesis in bladder carcinoma based on counting in hot spots have been published (Table 5

**Table 5:**
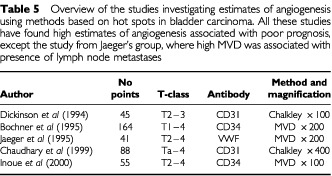
Overview of the studies investigating estimates of angiogenesis using methods based on hot spots in bladder carcinoma. All these studies have found high estimates of angiogenesis associated with poor prognosis, except the study from Jaeger's group, where high MVD was associated with presence of lymph node metastases

). Four of these reported high vascular density to be associated with poor prognosis (Dickinson *et al*, 1994; Bochner *et al*, 1995; Chaudhary *et al*, 1999; Inoue *et al*, 2000) whereas one showed high vascular density to be associated with classical prognostic factors at diagnosis (Jaeger *et al*, 1995). None of the studies mentioned the possible influence of inflammation in the carcinomas and how to deal with it when identifying the area of highest vascular density. In contrast to the present study, which investigated tissue removed during a TUR-B before cystectomy, Bochner *et al* (1995) investigated tissue from cystectomies of presumably consecutive patients. In our experience the latter tissue is typically more inflamed than tissue from a TUR-B. In the study by Dickinson *et al* (1994), tissue from TUR-B on 45 patients was used. Although inflammation was not mentioned, we judge from the colour illustration in the paper that estimates of angiogenesis were performed also in invasive tumour areas with signs of moderate inflammation. Their study investigated the prognostic value of estimates of angiogenesis in bladder carcinoma, however, they did not state the prognostic value of their Chalkley counts dichotomised on the median value, but used an ‘optimal cutpoint’. The study by Jaeger *et al* (1995) on 41 patients correlated estimates of tumour angiogenesis with lymph node metastases; no follow-up was reported. The most recent study identified high MVD as an independent parameter of poor prognosis in cystectomy specimens but not in TUR-B specimens. However, this was based on 55 patients only (Inoue *et al*, 2000). None of the studies mentioned in Table 5 used the same counting area as we did, therefore the cut-off points introduced in the studies are not directly comparable with the present study. Two of the studies in Table 5 used the Chalkley grid, which provides a kind of area estimate. In the current study we have also used the Chalkley grid as previously described (Offersen *et al*, 2001), and the Chalkley scores revealed the same results and led to the same conclusions as the MVD scores have done (data not shown). However, we preferred to report only the MVD method in this type of carcinoma, because the Chalkley scores, being a kind of area estimate, could in theory be influenced by vasodilation caused by the inflammatory cells.

VEGF is one of the key regulators of the angiogenic processes, therefore, we decided to evaluate the staining of this factor. The antibody used reacts with the 121, 165, and 189 amino acid splice variants; VEGF_121_ is freely diffusible, VEGF_165_ is bound both on cell surfaces and in the extracellular matrix (ECM) as well as being secreted, and VEGF_189_ is almost completely sequestered in the ECM (Ferrara, 1999). The receptors for VEGF are of the tyrosine kinase family (VEGFR-1, VEGFR-2 and VEGFR-3) and are largely restricted to vascular endothelial cells (Ferrara, 1999; Yancopoulos *et al*, 2000). However, evidence exists that VEGFR-1 is also transiently located in a subgroup of highly differentiated prostate carcinoma cells (Hahn *et al*, 2000) and on macrophages (Sawano *et al*, 2001). To our knowledge no reports have demonstrated the localisation of VEGF receptors on inflammatory cells, therefore, the intense staining of the inflammatory cells in this study represents genuine VEGF production in these cells. Indeed, by demonstrating VEGF mRNA in T cells infiltrating human bladder and prostate carcinoma by *in situ* hybridisation, Freeman *et al* (1995) suggested that lymphocytes play an active role in the regulation of angiogenesis, not associated with other immune reactions but driven by VEGF. Hypoxia is the primary stimulator of VEGF synthesis (Ferrara, 1999), but it has lately been demonstrated that release of VEGF from neutrophils may occur independently of hypoxia (Koehne *et al*, 2000). Taken together, this suggests that the ‘host factor’ has to be taken into account when evaluating bladder carcinomas. For example, in a study by O'Brien *et al* (1995) different angiogenic pathways are suggested based on measurements of VEGF using a RNase protection analysis. They found the VEGF content in superficial tumours four-fold higher than in the invasive tumours and 10-fold higher than in normal bladder tissue, but these measurements may be influenced by VEGF from host cells. Recently, another group has tried to measure the relative contribution to VEGF production of host *vs* tumour cells (Tsuzuki *et al*, 2000). VEGF^−/−^ and wild-type embryonic stem cells were implanted in severe combined immunodeficient mice, and VEGF ELISAs showed that in the VEGF^−/−^ embryonic stem cell-derived tumours the content of VEGF was about half that in the wild-type derived tumours, the host cells contributing the other half.

To our knowledge, inflammation has not been described as an independent prognostic factor in invasive bladder carcinoma, although pronounced lymphocytic infiltration at the advancing tumour front was identified by Jass (1986) as an independent prognostic parameter in rectal adenocarcinomas. The survival advantage we describe may reflect a host versus tumour response, as also suggested by Jass, but this will require further analysis for confirmation.

Most of the tumours included in the present study were muscle invasive, and it was shown that malignancy grade was not a significant independent variable. Previously, malignancy grade has been established as an independent prognostic variable in non-invasive bladder tumours, however, as stated in a WHO consensus classification of urothelial neoplasms ‘invasive tumours should be graded as low or high’ (Epstein *et al*, 1998), since there is no difference in the outcome of patients diagnosed with muscle-invasive tumours of either grade III and IV. For example, Jimenez *et al* (2000) investigated the prognostic potential of malignancy grading in 93 muscle-invasive bladder tumours and found no difference in outcome between grade III and IV, and similar results were seen in the study by Bassi *et al* (1999) who investigated 369 bladder tumours, where 258 (70%) were muscle-invasive: malignancy grade was not an independent variable. In a study from Bostwick's group (co-author on the WHO consensus classification) malignancy grade III and IV a.m. Bergkvist are classified together as ‘high grade’ (Cheng *et al*, 2000). Thus, it is not surprising that we found no difference in the prognosis between patients diagnosed with grade III and IV muscle-invasive tumours.

In the current study 67 patients (63%) underwent radical cystectomy and 40 patients (37%) received radio- and/or chemotherapy. As systemic therapy was given to patients with advanced disease, the outcome of these patients was strongly influenced primarily by T and N status. There was no difference in the outcome of the patients in multivariate analysis with regard to cystectomy or chemo- or radiation therapy. Only eight cases in our study had a history of BCG instillation of the bladder, two of them had tumours with intense inflammation somewhere in the carcinoma. Typically, after BCG instillation, germinal centres are seen quite soon, and then tend to disappear. However, germinal centres were not considered in this study.

Our findings reveal a significant association between increasing degrees of inflammation and rising estimates of angiogenesis. High degree of inflammation were identified as an independent prognostic factor using death of disease as the endpoint, the relative risk being 0.48 when areas of intense inflammation were present. When evaluating MVD irrespective of inflammation a significantly better overall and disease-specific survival was found for patients with tumours of high vascular density. MVD_max_ and degree of inflammation was closely associated, and using overall death as the endpoint identified MVD_max_ as an independent parameter, while inflammation lost importance. Using VEGF stained sections we demonstrated both carcinoma and host inflammatory cells as contributors of VEGF to the tissues. The fact that inflammation in bladder carcinoma indicates a good prognosis may reflect the ability of the patient to mount an immune response. As seen in Figure 3, the areas of intense inflammation show the immune cells in direct contact with the carcinoma cells. Our findings also point towards angiogenesis being associated with inflammation in bladder carcinoma and being regulated by a delicate balance between factors from both the tumour and the patient. Previous research on angiogenesis has been based solely on the carcinoma cells as contributors of different angiogenic factors (Hanahan and Folkman, 1996), however, our data show that reactive cells take an active part in the stimulation of angiogenesis and, therefore, need to be taken into account in the design of future angiogenic strategies.
